# Cisplatin and Short-Term 5-Fluorouracil Infusion for Paraneoplastic Microangiopathic Hemolytic Anemia in Gastric Cancer: A Case Report and Review of the Literature

**DOI:** 10.1155/2013/594787

**Published:** 2013-12-29

**Authors:** M. S. Sanatani, A. Lazo-Langner, I. M. Al-Rasheedy

**Affiliations:** ^1^Department of Medical Oncology, Western University, 790 Commissioners Road East, London, ON, Canada N6A 4L6; ^2^Department of Medicine, Division of Hematology, Western University, 800 Commissioners Road E E6-216, London, ON, Canada N6A 5W9; ^3^Department of Oncology, Western University, 800 Commissioners Road E E6-216, London, ON, Canada N6A 5W9; ^4^Department of Epidemiology and Biostatistics, Western University, 800 Commissioners Road E E6-216, London, ON, Canada N6A 5W9; ^5^Medical Oncology, King Abdulaziz Medical City, Princess Noorah Oncology Center, Jeddah, Saudi Arabia

## Abstract

Microangiopathic hemolytic anemia is a rare paraneoplastic syndrome accompanying adenocarcinoma of the stomach. We report on a patient presenting with anemia due to a combination of severe hemolysis and tumour bleeding, where the combination of cisplatin and 5-fluorouracil in a short course infusional regimen led to a complete response of the hematologic abnormalities in the first line setting. Relapse was successfully treated with second line docetaxel; however the response was relatively short-lived. Overall survival was 16 months from diagnosis, which compares favourably to the survival of other reported cases. The chemotherapy regimens used in previously reported similar cases are reviewed. We suggest that a regimen based on bolus 5-fluorouracil, possibly with a platinum, should be investigated as a possible regimen of choice.

## 1. Introduction

Cancer-associated microangiopathic hemolytic anemia (MAHA) is a rare, potentially fatal complication of malignant tumors, usually associated with poor prognosis. It has been reported to occur in gastric, breast, prostate, and lung cancer and also a few cases have been reported in patients with carcinoma of unknown origin [1]. This condition was first described by Brain et al. in 1962 [2]. It is defined as a severe hemolytic anemia in the setting of malignancy with negative Coombs' test and fragmented red blood cells in the peripheral blood smear. The clinical presentation may include features of DIC (disseminated intravascular coagulation), TTP (thrombotic thrombocytopenic purpura), or HUS (hemolytic uremic syndrome) and the distinction between these conditions is usually not straightforward. We report on a case of a patient with severe hemolytic anemia in the setting of metastatic gastric cancer who experienced a complete resolution of the anemia and a marked tumor response after treatment with palliative chemotherapy. Building on a recently published review by Lechner and Obermeier [3], we also review prior reported cases of hemolysis in gastric cancer specifically in regard to the chemotherapy regimen used.

## 2. Presentation and Diagnosis

**Figure 1:**
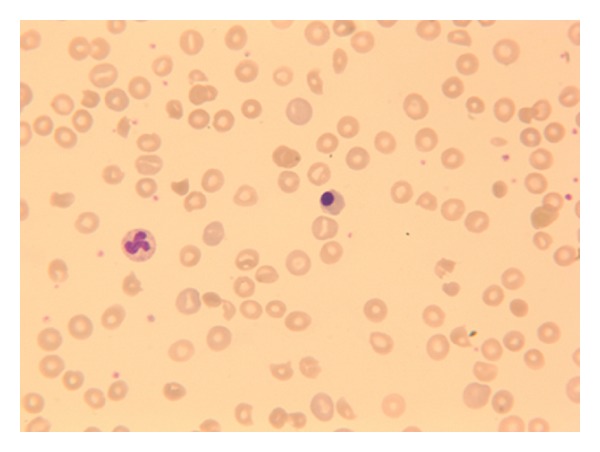
Polychromasia, nucleated red blood cells, and fragments on presentation.

**Figure 2:**
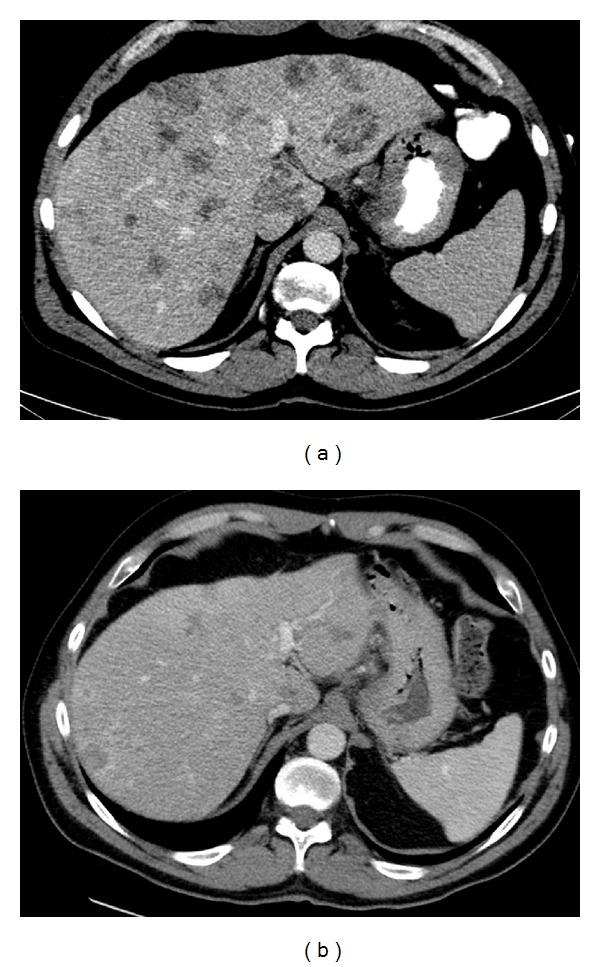
(a) Computed tomography imaging at presentation. (b) Computed tomography imaging after three cycles of cisplatin/5-FU.

A 49-year-old previously healthy male was admitted to the hospital with a history of increased dyspnea and fatigue over the course of several weeks. Other history was negative apart from a history of longstanding heartburn that was controlled with proton pump inhibitors and a history of early satiety for the last 1-2 years. There was no family history of malignancy. He was diagnosed with severe anemia (hemoglobin 52 g/L) and his peripheral smear showed schistocytes and polychromasia, as well as nucleated red blood cells (Figure 1). Serum free haptoglobin level was undetectable. Coombs' test was negative, bilirubin and LDH were elevated (73.1 *μ*mol/L [normal < 17.1] and 917 U/L [normal < 225], resp.), and a diagnosis of MAHA was made. In order to rule out an underlying solid tumor, CT scan of the chest, abdomen, and pelvis was performed demonstrating widespread metastatic disease, with thickening of the stomach and a GE junction mass measuring about 6 × 3.2 cm (Figure 2(a)). Gastroscopy was done and confirmed a gastroesophageal junction mass, with the biopsy showing low grade adenocarcinoma.

## 3. Initial Treatment Course

The patient was given transfusions, but his hemoglobin remained persistently below 80 g/L. He also developed episodes of melena. His laboratory tests showed worsening hemolysis, and after a drop in the platelet count (from 169,000/*μ*L on admission to 76,000/*μ*L after one week) a diagnosis of possible thrombotic thrombocytopenic purpura (TTP) was made and he was started on a trial of daily plasmapheresis without improvement.

Because of the melena, the patient was referred to radiation oncology and was treated with palliative radiotherapy (2000 cGy in 5 fractions) to the primary tumour in the stomach with resolution of the melena, but with no improvement in the transfusion requirements. A decision was made to start palliative chemotherapy with cisplatin and 5-fluorouracil (5-FU). A short intense regimen consisting of cisplatin 50 mg/m^2^ day 1 and 5-FU infusion 1000 mg/m^2^/day, days 1 through 4, all q21 days, was chosen to minimize days without plasmapheresis. However, during the first cycle plasmapheresis was discontinued due to lack of response, and shortly after the patient's hemoglobin started to stabilize with decreasing transfusion requirements and by three weeks after therapy start, he was no longer transfusion dependent. He was discharged from hospital approximately one month after admission, ambulatory and with improving performance status.

The patient continued on chemotherapy with cisplatin/5-FU every three weeks as outlined above. After three cycles of chemotherapy, he was completely asymptomatic (ECOG 0), no longer requiring any transfusions. A CT scan (Figure 2(b)) showed a decrease in the number and size of liver metastases. He completed 10 cycles, after which the treatment was stopped because of worsening sensory neuropathy.

**Figure 3:**
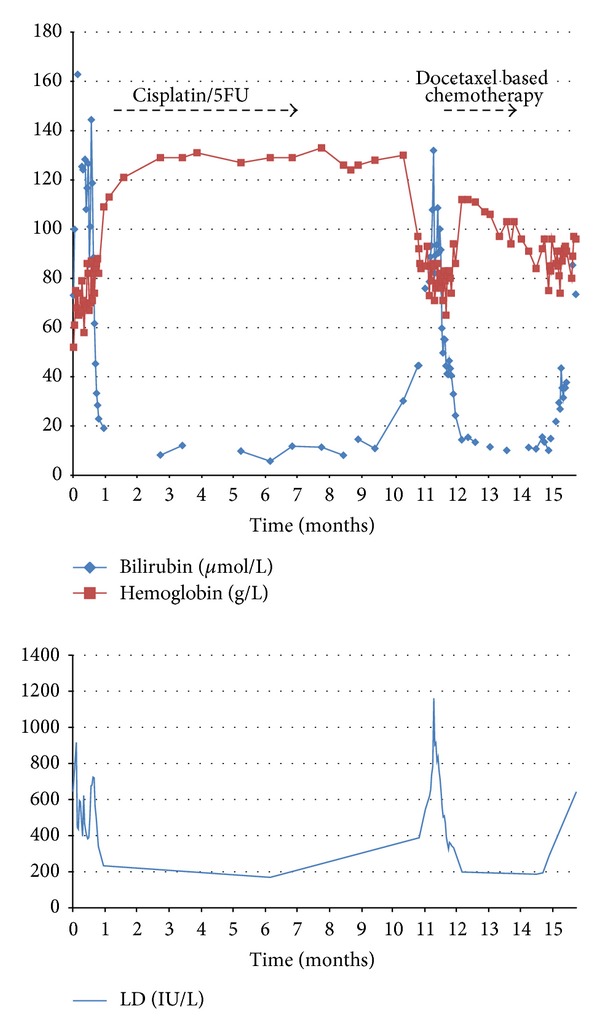
Hemoglobin, total bilirubin, and lactate dehydrogenase levels (U/L).

Two months after this last chemotherapy the patient presented on followup with progressive disease in form of recurrent hemolysis and worsening liver metastases, retroperitoneal lymphadenopathy, and a recurrent mass in the GE junction as well as melena. He received a single fraction of palliative radiation to the stomach and was started on second line chemotherapy with DCF (docetaxel 25 mg/m^2^ weekly, cisplatin 60 mg/m^2^ q21 days, and 200 mg/m^2^ 5-FU infusion). The hemoglobin and other indicators of hemolysis stabilized again (Figure 3), and CT scan followup after 3 cycles showed mixed response in the hepatic metastases. He continued the DCF chemotherapy for control of the hemolysis for a total of 6 cycles, with cisplatin and 5-FU then omitted due to declining performance status and mucositis (change to single agent docetaxel), but presented later with an intractable GI bleed from the primary tumour. Despite endoscopic intervention by the gastroenterology service he continued to deteriorate and comfort measures were instituted. He died of gastric hemorrhage, 16 months from the time of his initial presentation with stage IV gastric adenocarcinoma.

## 4. Discussion

Microangiopathic hemolytic anemia (MAHA) can be the first manifestation of malignancy such as gastroesophageal cancer. Indeed, several case reports [4–6] exist describing curative-intent surgery after diagnosis of an early stage gastric cancer presenting with MAHA. We report on the case of a previously undiagnosed gastroesophageal cancer patient with severe hemolytic anemia requiring up to 5 units daily of blood, in whom chemotherapy resulted in a complete response of the hemolysis and an ongoing response in the tumor burden for over a year. We chose a cisplatin and short-term infusion 5-FU regimen rather than the usual DCF or ECF (epirubicin/cisplatin/5-FU) treatments based on prior case reports [7, 8] and also initially to allow plasmapheresis, which however ultimately was abandoned quickly. We also had concerns initially regarding myelosuppression by a 3-agent regimen. Since the tumour was Her2neu negative no trastuzumab therapy was considered.

Published cases of MAHA associated with gastric cancer [3] are detailed in Table 1 [4–36].

Two observations from this summary are worth pointing out.

*(1) Diagnosis and Purported Mechanism.* There are many reported cases of hemolytic anemia with or without thrombocytopenia in gastric cancer. However, a unifying diagnosis or nomenclature remains elusive. In several cases (including ours) clinical consideration was given to the diagnosis of TTP/HUS. As in many other published cases of CR-MAHA, plasmapheresis was performed; however it did not seem to have a significant effect. Carr et al. [13] have reported on a patient with gastric cancer and TTP where plasmapheresis did improve gastrointestinal bleeding, fever, and mental status changes; however that patient's presentation may have had a different pathophysiological basis than our case. All these observations raise the question of whether malignancy-associated MAHA that is unresponsive to plasmapheresis is a form of TTP/HUS or rather is a completely different disease entity and although some reduction in ADAMTS13 metalloproteinase activity has been reported in cancer patients with hemolysis, this can also be the case in other clinical settings [37]. Levels of von-Willebrand factor cleaving protease are not consistently altered in CR-MAHA associated with thrombocytopenia [12]. Future research into the pathophysiology underlying CR-MAHA, as well as clarification of nomenclature, classification, and distinction between TTP, HUS, DIC, and possibly “CR-MAHA with thrombocytopenia NOS (not otherwise specified),” is urgently required.

*(2) Heterogeneity of Management Approaches*. A review of the published cases highlights the heterogeneity of the treatment approaches used for patients presenting with hemolysis and gastric cancer and reflects the evolution of systemic therapy for this malignancy over the last three decades. Cytotoxics used, in various combinations, were carboplatin, cisplatin, oxaliplatin, paclitaxel, docetaxel, etoposide, 5-fluorouracil, adriamycin, mitomycin-C, vincristine, methotrexate, cyclophosphamide, gemcitabine, irinotecan, and hydroxyurea. Interestingly, only very few publications [7, 8, 26] provided all the details of the chemotherapy regimen required to prescribe it. Many patients apparently did not receive chemotherapy because of poor performance status. Based on our experience we would suggest that gastric cancer patients with CR-MAHA may benefit from a trial of chemotherapy, within reason, even though they may appear too ill for this. Our patient had an ECOG (Eastern Cooperative Oncology Group) performance status of 4 when chemotherapy was started but improved to ECOG 0 within a few months. Approximately one quarter of the patients in this review received plasmapheresis, with only one patient deriving clinical benefit [13].

*(3) Is There an “Anecdotally Best” Treatment Approach?* Based on our case as well as the review of other cases, we would suggest several management principles for future cases. In regard to diagnosis, to avoid confusion, an overall inclusive diagnostic term for a condition presenting with evidence of hemolysis, negative Coombs' test, thrombocytopenia, gastric malignancy known or suspected, and absence of renal failure or mental status changes may be “gastric-cancer-associated MAHA with thrombocytopenia.” On establishing that diagnosis, it would appear that rapid initiation of chemotherapy is of greater importance than that of plasmapheresis. This may not apply in cases where the presentation has most of the features of classical TTP or HUS including hemorrhage or renal or nervous system dysfunction.

Second, it would appear that chemotherapy regimens including cisplatin as well as bolus 5-fluorouracil (our patient, [7, 8, 23, 31]) may be the treatment of choice as anecdotally patients treated with such regimens had superior survival in the cases reviewed here. Third, chemotherapy should perhaps be continued in situations where it usually would not be, (e.g., despite developing adverse events and tumour growth). In retrospect, even though our patient eventually did have a relatively long survival, it is conceivable that continuing chemotherapy despite the worsening neuropathy could have had a beneficial effect on preventing the recurrence of the hemolysis. We also demonstrated control of hemolysis in the setting of progressive tumour growth on second line chemotherapy, although the duration of control was much shorter in this setting (6 versus 10 cycles on first line therapy with ongoing tumour control).

Further research into the etiology and mechanism of malignancy-associated MAHA and its connection to TTP/HUS, as well as development of consistent terminology and diagnostic criteria, may shed some further light on management of similar cases in the future.

## Figures and Tables

**Table 1:** 

Reference	Age	M/F	Hb (g/L)	Platelets (×10^9^/L)	Plasma-pheresis	Hematological diagnosis	Chemotherapy 1st line	Subsequent chemotherapy	Approximate survival from the beginning of the first therapy for hemolysis (if applicable) or presentation
[9]	70	M	72	80	Yes	TTP	Carboplatin/paclitaxel		14 days
[10]	52	F	72	62	No	MAHA	None		1 week
[10]	28	M	75	77	No	MAHA	None		1 week
[10]	21	M	61	342	No	MAHA	None		1 week
[8]	62	M	107	86	No	MAHA	Etoposide 120 mg/m^2^, leucovorin 300 mg/m^2^, and 5-FU 500 mg/m^2^ days 1–3, every 21 days	5-FU 2000 mg/m^2^, leucovorin 500 mg/m^2^, oxaliplatin 130 mg/m^2^ day 1 every 14 days	12 months
[11]	19	M	100	98	No	MAHA with DIC	5-FU, adriamycin, and mitomycin-C		6 days
[12]	61	F	95 est	NR	No	Cancer-related thrombotic microangiopathy	Infusional 5-FU 250 mg/m^2^/day		35 days
[13]	60	M	82	43	Yes	TTP	5-FU, adriamycin, and mitomycin C		6 months
[14]	59	F	61	20	Yes	TTP	None		1 month
[14]	65	F	80	86	No	TTP	Paclitaxel		11 months
[15]	52	M	60	24	Yes	MAHA without DIC	None		4 weeks
[16]	75	M	81	NR	No	MAHA	None		20 days
[17]	58	F	58	14	No	MAHA	5-FU, vincristine, methotrexate, and cyclophosphamide		10 days
[18]	50	M	73	50	Yes	Cancer-associated MAHA	Docetaxel, 5-FU, and platinum		12 days
[4]	71	M	44	18	No	TTP	None		Curative-intent surgery
[19]	25	F	74	100	No	MAHA	5-FU 600 mg/m^2^ weekly + adriamycin 40 mg/m^2^ day 1, q21 days	Mitomycin-C 10 mg/m^2^ monthly	7 months
[20]	28	F	52	111	Yes	TTP	Cisplatin/5-FU infusion		Under 2 months
[7]	45	M	59	50	No	MAHA and thrombocytopenia	Cisplatin 60 mg/m^2^ day 1, 5-FU 600 mg/m^2^/day infusion days 1–4, every 21 days		7 weeks
[7]	32	F	NR	NR	Yes	MAHA and thrombocytopenia	Cisplatin 20 mg/m^2^ days 1–5, etoposide 60 mg/m^2^ days 1–5, every 21 days	5-FU 298 (=70% of 425) mg/m^2^ and leucovorin 20 mg/m^2^ days 1–5, and weekly cetuximab 500 mg IV, all every 21 days	12 weeks approx..
[5]	71	F	56	15.6	No	MAHA	Mitomycin-C 10 mg and neothramycin (also gabexate infusion), 5 doses over 2 months		3 months
[6]	69	M	65	64	Yes	Thrombotic microangiopathy with renal failure	N/A		Curative intent surgery
[21]	60	M	98	35	No	DIC	None		24 days
[22]	83	F	40	85	No	MHA	None		3 days
[23]	47	F	49	9	No	MAHA	Cisplatin 80 mg/m^2^ day 1, 5-FU 1000 mg/m^2^/day, days 1–5	FOLFIRI, DCF at 20% dose reduction	19 months
[24]	69	F	96	104	No	MAHA	Adriamycin		4 weeks
[25]	44	M	74	71	No	MAHA and DIC	None		3 months
[26]	57	M	58	39	No	DIC	Methotrexate 100 mg/m^2^ day 1, 5-FU bolus 600 mg/m^2^ day 1, LV 10 mg/m^2^ q6h days 2 and 3, weekly		10 months
[27]	52	F	68	18	No	Thrombotic microangiopathy	5-FU, cisplatin, and hydroxyurea		3 months
[27]	51	M	74	74	No	Thrombotic microangiopathy	Gemcitabine, oxaliplatin, irinotecan, 5-FU, and docetaxel (details not reported)		4 months
[27]	59	M	66	39	Yes	Thrombotic microangiopathy	None		44 days
[28]	51	F	67	40	Yes	TTP	Vincristine 2 mg	Cisplatin 50 mg/m^2^days 1 + 15, 5-FU 150 mg/m^2^, and LV 500 mg weekly	3 months
[29]	59	F	86	23	No	DIC	None		10 days
[29]	51	F	39	120	No	DIC	None		22 days
[29]	46	F	115	59	No	DIC	None		8 days
[30]	43	M	53	NR	No	HUS	None		Surgery, no recurrence
[31]	66	M	70	250	No	MAHA	5-FU 425 mg/m^2^ and leucovorin 20 mg/m^2^ daily	5-FU 425 mg/m^2^ and leucovorin 20 mg/m^2^ daily	26 months
[32]	NR	NR	43	45	No	MAHA	Cisplatin/5-FU		2 days
[33]	50	M	85	95	No	Pulmonary tumour Thrombotic microangiopathy	5-FU 250 mg daily, mitomycin C 7 mg weekly, and cisplatin 125 mg once		86 days
[34]	66	M	73	28	No	Hemol. anemia	None		15 days
[34]	43	M	91	50		Hemol. anemia	None		7 days
[35]	43	M	78	62	No	DIC/MAHA	MMC, 5-FU, Ara-C, and heparin		6 months
[36]	27	F	72	23	Yes	Thrombotic microangiopathy	None		3 days
Our case	49	M	52	76	Yes	CR-MAHA	Cisplatin 50 mg/m^2 ^day 1, 5-FU infusion 1000 mg/m^2^/day, days 1 through 4, all every 21 days	Docetaxel 25 mg/m^2^ weekly, cisplatin 60 mg/m^2^ every 21 days, and 200 mg/m^2 ^5-FU infusion daily	16 months

CR: cancer-related; DIC: disseminated intravascular coagulation; 5-FU: 5-fluorouracil; HUS: hemolytic uremic syndrome; MAHA: microangiopathic hemolytic anemia; MMC: mitomycin-C; TTP: thrombotic thrombocytopenic purpura.
